# Audit of delays in the diversion of mentally disordered defendants under the Mental Health Act 1983/2007 at a liaison and diversion service in North West London

**DOI:** 10.1192/bjo.2021.932

**Published:** 2021-06-18

**Authors:** Seema Sukhwal, Claire Gordon-Ellis, Luneta Tajblova

**Affiliations:** 1 Barnet Enfield and Haringey MH NHS Trust; 2 Central and North West London NHS Foundation Trust; 3 Together for Mental Wellbeing

## Abstract

**Aims:**

To ascertain the length of time defendants wait for a Mental Health Act assessment (MHAA) and where necessary, how long they are waiting for a hospital bed.

**Background:**

The Liaison and Diversion Service in North West London (the Service) is provided by Central North West London Foundation NHS Trust (CNWL), Barnet Enfield Haringey (BEH) and Together to Willesden Magistrates Court in North West London.

One of the core activities of the Service is diverting individuals from the criminal justice system to hospital under the Mental Health Act (MHA).

The Code of Practice allows for a period of 14 days between the medical recommendation and conveyance to hospital. Defendants needing admission under MHA are remanded to custody if a bed is not available. This prevents them from receiving the assessment and care they need. We consider that all defendants found to be liable to detention under the MHA should be admitted to a hospital bed on the same day.

**Method:**

Data were collected between October 2018 and February 2019. All patients referred for a MHAA were included. The time a MHAA was requested, took place as well as how long the defendant waited for a bed was noted.

**Result:**

A total of 42 MHAA were requested. 25 individuals were detained under Section 2 of the MHA 1983.

The time between referral for a MHAA and the MHAA taking place was obtained in 25 of the 42 referrals. The range of times between a referral being made and the assessment taking placed varied between 1.5 hours and 22 hours. Two defendants were remanded overnight in prison as the MHAA could not take place on the same day as the referral.

In the 25 cases where an application for detention under Section 2 of the MHA was made, beds were not available on the same day in 7 cases. In 4 cases defendants required remand in prison custody due to beds not being available.

**Conclusion:**

There were some limitations to this audit as data were not available for all 42 individuals referred for a MHAA.

Individuals referred for MHAA by the Service had both medical recommendations completed within 5 days and those who required admission to hospital were admitted within 14 days of the recommendations being completed.

Whilst these standards are being met, individuals referred for MHAA and those requiring admission to hospital are still facing remand to custody.

